# Room temperature single-step synthesis of metal decorated boron-rich nanowires via laser ablation

**DOI:** 10.1186/s40580-019-0185-2

**Published:** 2019-05-08

**Authors:** Ignacio G. Gonzalez-Martinez, Alicja Bachmatiuk, Thomas Gemming, Gianaurelio Cuniberti, Barbara Trzebicka, Mark H. Rummeli

**Affiliations:** 1 Centre of Polymer and Carbon Materials, Polish Academy of Sciences, M. Curie-Sklodowskiej 34, 41-819 Zabrze, Poland; 2 Leibniz Institute for Solid State Research Dresden (IFW Dresden), Helmholtz Strasse 20, 01171 Dresden, Germany; 3 Institute for Materials Science and Max Bergmann Center of Biomaterials, TU Dresden, 01062 Dresden, Germany

**Keywords:** Decorated nanowires, Nanoparticles, Laser ablation, Room temperature, Single step synthesis

## Abstract

Hybrid nanostructures, such as those with nanoparticles anchored on the surface of nanowires, or decorated nanowires, have a large number of potential and tested applications such as: gas sensing, catalysis, plasmonic waveguides, supercapacitors and more. The downside of these nanostructures is their production. Generally, multi-step synthesis procedures are used, with the nanowires and the nanoparticles typically produced separately and then integrated. The few existent single-step methods are lengthy or necessitate highly dedicated setups. In this paper we report a single-step and rapid (ca. 1 min) laser ablation synthesis method which produces a wide variety of boron-rich decorated nanowires. Furthermore, the method is carried at room temperature. The synthesis process consists on a filamentary jet ejection process driven by pressure gradients generated by the ablation plume on the rims of the irradiation crater. Simultaneously nanoparticles are nucleated and deposited on the filaments thus producing hybrid decorated nanowires.

**Electronic supplementary material:**

The online version of this article (10.1186/s40580-019-0185-2) contains supplementary material, which is available to authorized users.

## Introduction

The nanotechnological revolution has been to a large extent fuelled by the fact that the properties of any given material can be drastically modified simply by down-scaling its extension along at least one of its dimensions. Thus, in addition to tailoring the properties of mater by engineering its chemical composition, nanotechnology offers the possibility of creating materials with novel properties merely by sculpting their morphology. When chemical heterogeneity and morphological variety are combined, it is possible to produce complex hybrid nanostructures with emergent properties that none of their individual components possess. Therefore, hybrid nanostructures can meet engineering challenges that less complex nanostructures cannot accomplish.

In particular, the group of hybrid nanostructures usually referred to as “decorated nanowires”, which are with nanoparticles (NPs) anchored on nanowires (NWs), has attracted a good deal of attention since they have a huge applicability potential due to their distinctive emergent properties. For instance, the NPs on various types of decorated NWs have enhanced photocatalytic activity that can be used for the degradation of dissolved dye molecules [1–5], exploited in conjunction with the modified conductometric properties to build gas-sensing devices [6–10]. Plasmonic resonances of the anchored NPs produce surface-enhanced Raman scattering signals that make it possible to collect Raman spectra of nanomolar concentrations of dissolved molecules [11–14] as well as producing enhanced photoluminescence emissions [15–20]. Dielectric nanowires decorated with metallic nanoparticles can be used to manufacture wavelength-controlled nanoswitches [21], refractive index sensors [22], supercapacitors [23], porous conducting solids [24] or interconnected structures with improved electrochemical properties [25]. More opto-electronic and sensing applications for decorated nanowires are described in a review by Pescagliani and Iacopino [26]. Clearly, there is no shortage of tested and potential applications for decorated nanowires. The shortcomings are to be found in their synthesis methods.

The vast majority of synthesis processes for decorated nanowires is laborious and time-consuming. This is because the available methods are multi-step processes. In general, there are steps to produce the NWs followed by subsequent steps to produce and put the decorating NPs on them.

Several methods involve the chemical etching of solid substrates to produce NWs over which NPs are subsequently deposited by: casting drops of a deposition solution over the NWs [13] or dipping the NWs into it [1, 5, 12], pulsed laser ablation [4, 13], specific chemical reactions [2, 14] or spin-coating [17]. The NWs as well as the NPs can be produced in sequential [25] or separate [7, 23] “wet” chemical processes. NWs can be produced through electro-chemical methods or seed-assisted chemical baths while the NPs form from annealed films previously sputtered onto nanowires [9] or by photochemical deposition [18].

Other alternatives are to produce the NWs/nanorods by the vapor–liquid–solid (VLS) mechanism and subsequently deposit the nanoparticles via vapor-deposition [10] or sputtering methods [6, 15, 16]. Methods based on solid–liquid–solid or oxide-assisted growth can also be used to produce NWs with NPs deposited via wet chemical procedures [3, 19].

Other multi-step methods involve: the production of NWs by plasma-assisted chemical vapor deposition followed by thermal deposition and intercalating annealing steps [22] or making gels containing exfoliated NWs to produce networks of interlinked decorated NWs after drying [24].

We could find only two single-step synthesis methods. The first is laborious since it needs the production of layered wafers to assemble a microreactor where the growth of the hybrid nanostructures is driven by microwave enhanced-plasma deposition [10, 21]. The second produces Au-peapod NWs by high temperature annealing (1030 °C) of an Au layer deposited on Si via ion-beam sputtering. The disadvantages being the high temperature needed and the relatively lengthy processing (10–30 min).

Since decorated NWs have a remarkably wide range of applicability, arguably, the main obstacle to their full inclusion into marketable devices is the lack of more straightforward synthesis methods. Therefore, here we report a one-step, room-temperature laser ablation procedure that can be easily adapted to produce a large variety of decorated nanowires. The method produces different types of boron-rich nanowires decorated by NPs by ablating solid pellets chiefly made of B_2_O_3_ with an added metal oxide and a small amount of a pure metal. The selection of the specific metal oxide determines important aspects of the morphology of the NWs while the chosen pure metal dictates the composition of the decorating NPs. The laser ablation procedure yields macroscopic amounts of NWs, however, the total yield is limited by the small size of the ablated pellets (around 3 mm of thickness and a diameter of nearly 13 mm). Additionally, our method is remarkably quick (ca. 1 min), this feature could make it attractive for industrial-scale mass production of decorated NWs if larger pellet targets can be accommodated in dedicated laser ablation setups.

## Materials and methods

**Fig. 1:**
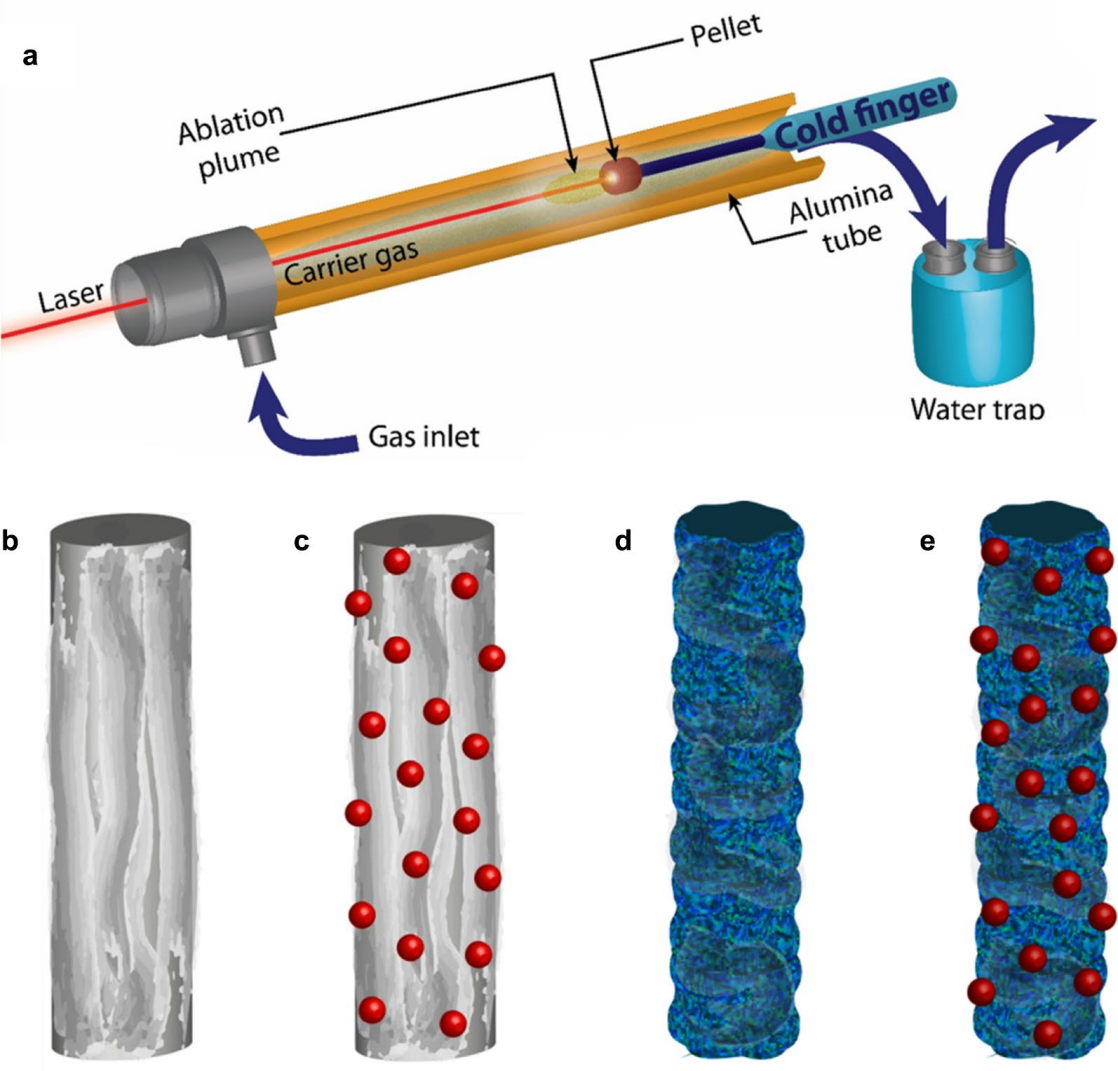
Overview of the laser ablation setup and the nanowires. **a** Schematic drawing of the laser ablation setup used to synthesize distinct types of boron-based nanowires. A pellet held by a holder connected to a cold finger is inserted into an alumina tube chamber while the laser enters the chamber at the other extreme. **b**–**e** Schematic drawings of the microstuctural morphology of the various types of nanowires produced in the setup in **a**. Four basic type of morphologies are observed: fibrous and “bare” (**b**), fibrous and decorated with nanoparticles (**c**), coarse and bare (**d**) and coarse and decorated with nanoparticles (**e**)

A schematic view of our laser ablation setup is depicted in Fig. 1a and it is also described elsewhere [27]. Briefly, a Q-switched Nd:YAG pulsed-laser (wavelength of 1064 nm, frequency of 10 Hz, pulse width of 8 ns, pulse power of 0.31 GW and fluence per pulse of 2.21 × 10^6^ J/cm^2^ since the irradiation spot has a radius of around 0.06 mm) is fired onto a compressed pellet held by a tantalum holder attached to a copper “cold finger” inside of which there is a constant flow of cold water. The cold finger with the holder and pellet are inserted into an alumina tube to create a closed chamber where the ablation reaction takes place. A steady stream of Ar at 1 l/min is flown into the alumina chamber which was always kept at atmospheric pressure. Each ablation round lasted for around 1 min. The final product consists on a fibrous sponge-like aggregate part of which was found hanging directly from the ablated target and part of it was collected by gently scrapping it from the cold finger.

The chemical composition of the ablated pellets varied depending on the kind of nanowire and decoration intended, however, there is a generic composition that holds true of all pellets. The main component is B_2_O_3_ (99.98% purity from Alfa Aesar) forming the basis of all pellets with a molecular percentage (mol.%) of at least 70. Then a selected metal oxide was added with a mol.% ranging from 15 to 20. Finally, a pure metallic element with a maximum mol.% of 1 was also added (the specific molecular ratios of each pellet can be found in Table 1 and the purities as well as the providers of all chemicals are in Additional file 1: Table S1). To produce the pellets, the mixture of powders in the desired molecular ratio was thoroughly grounded with a quartz mortar and pestle and then compressed in a hydraulic press at a pressure of 0.15 GPa.

**Table 1:** Summary of the distinctive characteristics of all the as-produced NWs

Pellet composition		Decorated	Not decorated	Average nanoparticle’s diameter (nm)	Diameter’s range (nm)	Coloration	Morphology	
Compounds/elements	Molecular ratio						Fibrous	Grainy
B_2_O_3_ + MgO	1:0.25		X			White	X	
B_2_O_3_ + MgO + Au	1:0.24:0.006	X		4.5	1.4–15.4	Pink	X	
B_2_O_3_ + MgO + Ag	1:0.24:0.006	X		3.9	2.0–8.6	Yellow	X	
B_2_O_3_ + MgO + Pd	1:0.24:0.006	X		5	1.1–30.8	Pale yellow	X	
B_2_O_3_ + MgO + Co	1:0.24:0.006	Few NPs		Too few		White	X	
B_2_O_3_ + MgO + Mo	1:0.24:0.006	Few NPs		5.9	2.1–16.9	White	X	
B_2_O_3_ + Mg0 + Cu	1:0.24:0.006		X			White	X	
B_2_O_3_ + ZnO	1:0.18		X			White	X	
B_2_O_3_ + ZnO + Au	1:0.42:0.0006	X		4.8	0.9–29.9	Pink	X	
B_2_0_3_ + ZnO + Ag	1:0.24:0.01	X		4.4	1.1–12.8	Yellow	X	
B_2_O_3_ + Ge + Au	1:0.24:0.006	X		6.4	1.1–24.2	–	X	
B_2_O_3_ + TiO_2_ + Au	1:0.24:0.006	X		4.4	1.3–14.4	Purple		X
B_2_O_3_ + TiO_2_ + Ag	1:0.24:0.006	Few NPs		Too few		Grey		X
B_2_O_3_ + MoO_3_ + Au	1:0.24:0.006	X		8.3	1.6–23.6	Purplish blue		X
B_2_O_3_ + MoO_3_ + Ag	1:0.24:0.006	Few NPs		6.8	2.3–11.2	Bluish grey		X
B_2_O_3_ + Al_2_O_3_ + Au	1:0.24:0.006	Few NPs		2.9	1.1–5.8	Bluish grey		X

The transmission electron microscope (TEM) observations were carried out in a FEI-Tecnai F30 operated at 300 kV. The EDS studies were performed inside the TEM using a Bruker Quantax spectrometer. Scanning electron microscope (SEM) observations were performed in a JEOL JSM 6510 device.

## Results

The laser ablation experiments produced macroscopic yields of bundled nanowires. After analyzing the various products in the TEM we could establish that the NWs have four basic different morphologies depending on the pellet composition. Figure 1b–e show schematic representations of the four morphological types. The first type consists on “bare” nanowires with no decorative nanoparticles and with a somewhat uneven or fibrous surface (Fig. 1b). The second type of nanowires is morphologically similar to the first but with metallic nanoparticles as decorations (Fig. 1c). The third type of NWs is also bare but with a rough or coarse surface (Fig. 1d) and finally there are coarse NWs decorated with metallic NPs (Fig. 1e). All of the analyzed NWs are amorphous.

EDS analysis of various nanowires show that, regardless of their morphology, the dominant spectral peaks correspond to the elements used for manufacturing the pellets from where the NWs were produced (Additional file 1: Figure S1).

**Fig. 2:**
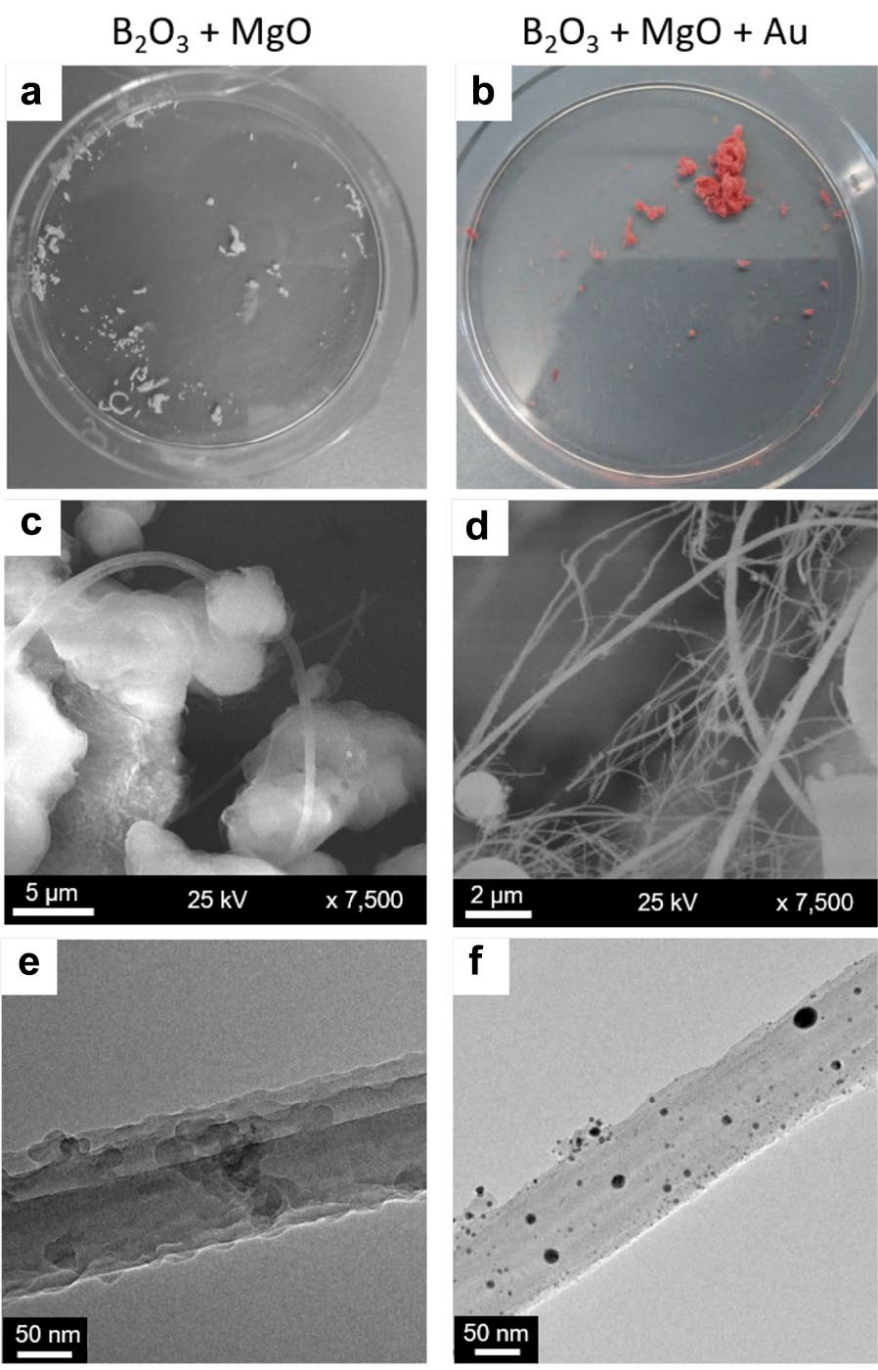
Comparison between non-decorated and decorated nanowires. Photographs of the products right after being collected from the cold finger, one from an ablated pellet containing B_2_O_3_:MgO (**a**) and one from a pellet containing B_2_O_3_:MgO:Au (**b**). Two differences can be noticed, when Au is added the yield increases and the product acquires a bright pink coloration. Additionally, the product on **b** has a fibrous sponge-like appearance. **c** A SEM micrograph of the product in **a**. Although it is possible to find nanowires, they are rarely found tangled up in large numbers. At least three nanowires can be seen in the micrograph. **d** SEM micrograph of the product in **b**. Here the nanowires are much more abundant. Bundles with numerous nanowires are easy to locate. **e** TEM micrograph of the product in **a**. The nanowires are amorphous. They have a slightly rough fiber-like morphology which can be identified as the morphological type depicted in Fig. 1a. **f** TEM micrograph of the product in **b**. The morphology of the nanowires is similar to the case without Au but here the nanowires are decorated with numerous nanoparticles which appear only when pure Au is added to the target. The morphology corresponds to the type depicted in Fig. 1b

Even prior to detailed characterization, there are two readily observable characteristics of the as-produced NWs: their coloration as well as their yield varies markedly depending on the pellet’s composition. Figure 2 shows the cases of NWs produced from pellets made of B_2_O_3_:MgO (molecular ratio 1:0.25) and B_2_O_3_:MgO:Au (molecular ratio 1:0.24:0.006) for comparison. The product from pellets without Au is white while that from pellets with Au is bright pink (Fig. 2a, b respectively). The NW yield of the former is lower in comparison from the latter, for instance, it is much harder to find nanowire bundles from B_2_O_3_:MgO ablated pellets when analyzing the product in the SEM, on the other hand dense bundles of NWs are routinely found in the products from B_2_O_3_:MgO:Au pellets (see Fig. 1c, d). On closer inspection, TEM micrographs reveal that the NWs from B_2_O_3_:MgO pellets are fibrous and not decorated while those from pallets where Au was added are fibrous and decorated with NPs.

**Fig. 3:**
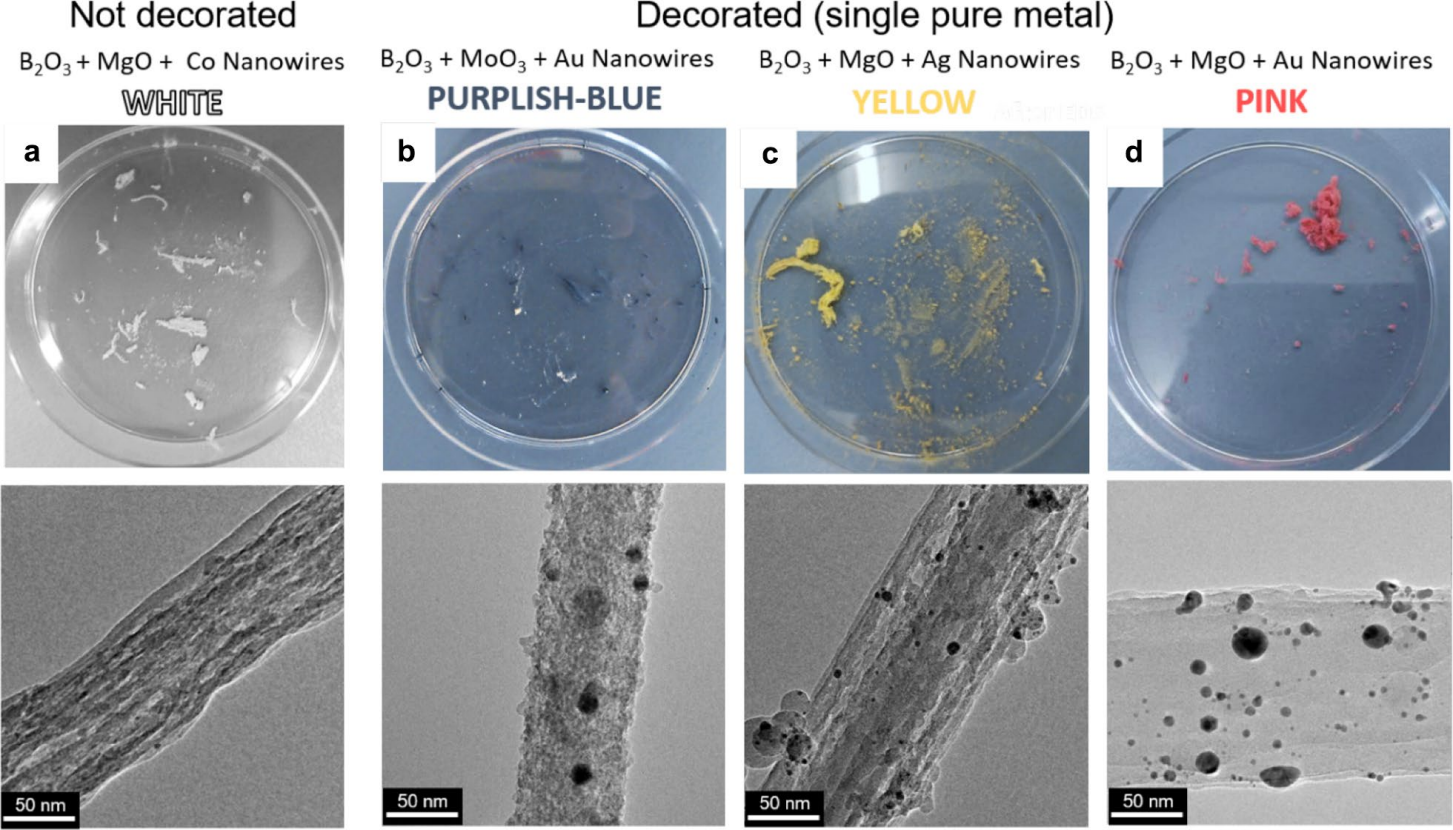
Nanowires with different colorations. The synthesis rounds tend to produce macroscopic amounts of nanowires with distinct colorations which depend on the composition of the ablated pellets. **a** A photograph of the white product from an ablated target containing B_2_O_3_:MgO:Co. The TEM micrograph below reveals that these nanowires are not decorated with nanoparticles. **b**–**d** Photographs of nanowires fabricated from targets containing, B_2_O_3_:MoO_3_:Au, B_2_O_3_:MgO:Ag and B_2_O_3_:MgO:Au, their colors are bluish gray, yellow and pink respectively. A TEM micrograph of each type of nanowire is right below each photograph. They all show that these nanowires are decorated with nanoparticles. By mixing more than one pure metal or two metal oxides and a pure metal in a single target one can obtain nanowires with new colorations. In general, macroscopic quantities of nanowires without decoration are white while decorated nanowires and some nanowires with coarse morphologies have distinctive colorations

Indeed, macroscopic amounts of white products contain only bare or poorly decorated NWs while colored products are made up primarily of large bundles of decorated NWs. This is true of almost all the combinations of chemical substances used to manufacture the pellets. For example, Ag-decorated nanowires tend to have a yellowish coloration while Au-decorated nanowires tend to be pink. There are some exceptions, some poorly decorated NWs containing TiO_2_, MoO_3_ or Al_2_O_3_ nanowires tend to have a bluish-grey coloration. These nanowires also happen to have a coarser/grainier surface morphology. Interesting cases are those of the NWs from pellets with B_2_O_3_:TiO_2_:Au and B_2_O_3_:MoO_3_:Au, in both cases the NWs are simultaneously well-decorated and have a coarse surface. Tellingly, their color is in the purple–blue side of the spectrum, which is what should be expected from mixing the bluish/purplish tone of all coarse NWs and the pink color of all the NWs decorated with Au NPs. Some examples of colored NWs can be seen in Fig. 3.

All in all, our single-step laser ablation technique produced sixteen different types of boron-rich nanowires. More than 80% of them were decorated to some degree. The average diameter of the NPs in all of the decorated NWs never exceeded 8.5 nm and it was of less than 5 nm in 66.6% of the cases. A summary of some of the most important chemical and structural characteristics of all the produced NWs is listed in Table 1.

The length of individual NWs could not be determined for a number of NWs that is sufficient to give a meaningful statistical figure. That is mostly due to the tightly tangled configuration of the as-produced NWs bundles which precluded us from tracking individual NWs in the SEM. On the other hand, the NWs were usually too long to be contained within the field of vision of the TEM working at its lowest possible magnitude making it impossible to measure their length in a reliable manner. However, few individual NWs could sporadically be found during the SEM observations and they typically had lengths ranging in the hundreds of microns.

**Fig. 4:**
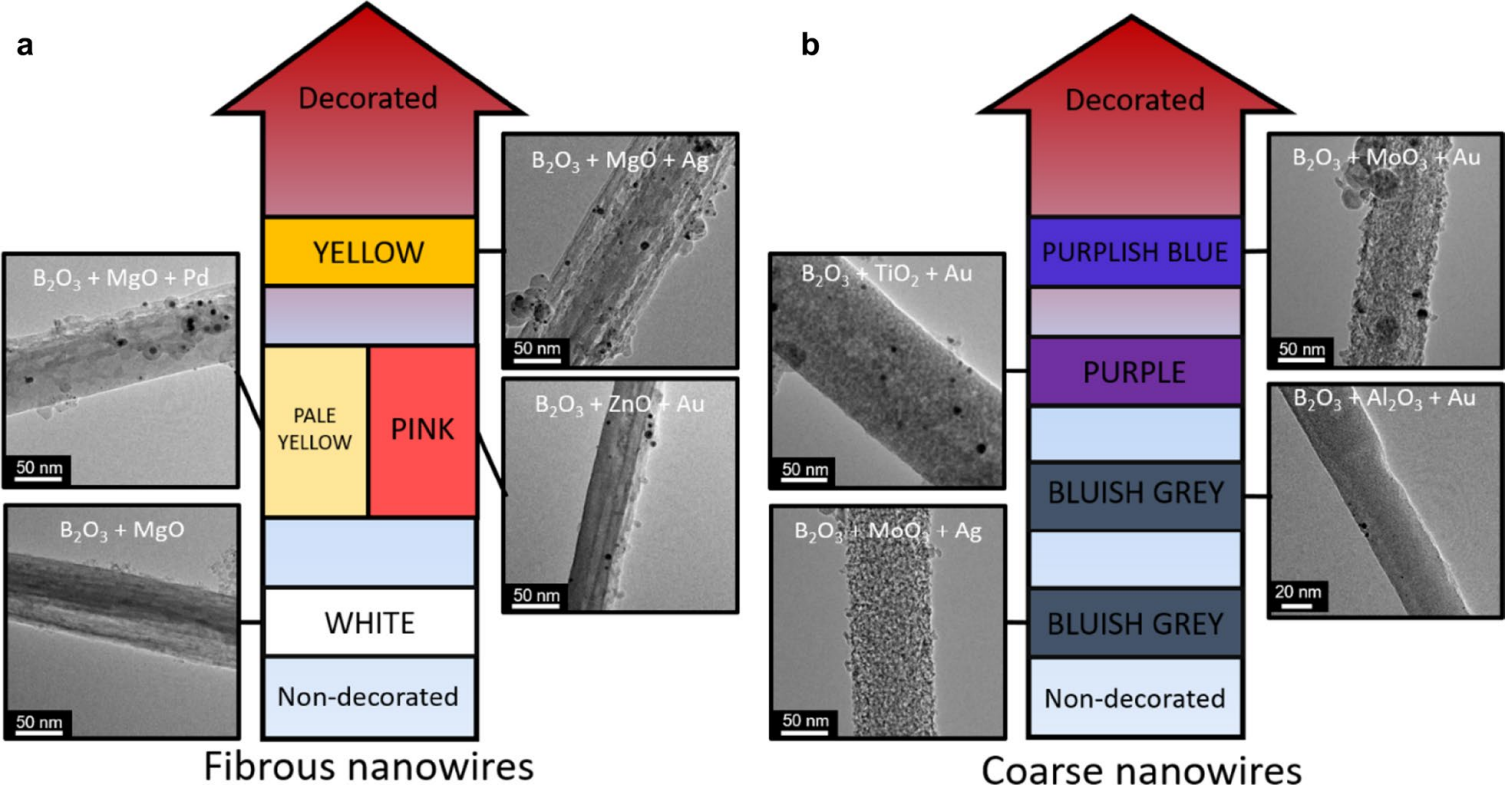
Degree of decoration of various nanowires. **a** Graphic representation of a qualitative scale of the degree of decoration of the fibrous nanowires. Four different types of nanowires have been selected as representative examples. Nanowires without decoration are placed near the base of the arrow as it is the case of B_2_O_3_:MgO nanowires. As the nanowires are gradually decorated with more nanoparticles they are placed higher in the arrow. The color of the boxes along the arrow matches the color of the macroscopic amounts of each type of nanowire. **b** An analogous type of graphic but for the case of nanowires with a coarse surface morphology

A more visual classification scheme based on the “degree of decoration” (which can be taken as an approximate average number of identifiable NPs per unit length) that also incorporates the coloration of some of the NWs can be appreciated in Fig. 4. An alternative visual classification scheme is shown in Additional file 1: Figure S2.

Another relevant aspect of the morphology of the NWs is the morphology of their tips. Typically, whenever NWs are produced by the laser ablation of targets with small amounts of a metallic element, the metal forms catalyst droplets that lead the growth of the NWs via the VLS mechanism [28]. When that happens, one usually finds a metallic NP at the tip of the NWs having a diameter commensurate with the NW diameter. However, all the NWs examined in the TEM lack metallic nanoparticles at their tips. Instead, the extremes of the NWs are either abruptly truncated (Additional file 1: Figure S3A–C) or they have a large bulbous end which seems to be continuous with the NWs rather than “attached” to them (Additional file 1: Figure S3D–F). This finding suggests that the NWs do not grow according to the VLS route or the related vapor–solid–solid (VSS) mechanism.

## Discussion

The primary focus of this work is to develop a simple and straightforward synthesis technique that bypasses the need of multiple steps to separately produce the individual components of the decorated NWs (as most of the existent synthesis processes require). This, of course, raises the question of why is it that our technique succeeds at producing complex hybrid nanostructures in a single step? The answer must be related to the growth mechanism of the hybrid nanostructures. The findings rule out the VLS and VSS mechanisms (Additional file 1: Figure S3) which are the typical catalytic mechanisms underlying the production of NWs via laser ablation, especially when pure metals are deliberately incorporated into the ablated target. Additional file 1: Figure S3 shows that the extremes of our NWs have two distinctive morphologies, they are either abruptly terminated or have a large bulbous extreme that seems to be continuous with, rather than attached to, the NWs.

Amorphous NWs (also known as “nanofibers”) with these and other kinds of tips have been repeatedly synthesized by pulsed laser ablation on polymers [29], silica glass [30–32], alkali-free glass [33], chalcogenide glass [34] as well as borosilicate glass [31]. Their morphology can be explained in terms of their growth mechanism which is largely equivalent in all cases. Briefly, the growth mechanism of the NWs can be described as a filamentary jet ejection process where the NWs form due to pressure gradients caused by the displacement of molten mass in the irradiated spot.

Venkatakrishnan et al. explain that rapid consecutive laser pulses generate heat in the irradiated spot which cannot be entirely diffused throughout the material, thus leading to heat accumulation that locally raises the temperature up to around 3000 °C, i.e. well beyond the melting point of the target [32]. This creates a molten area resembling a crater where hot and highly mobile material is produced. The molten material is displaced outwards due to the pressure exerted by the expanding plasma of the ablation plume. As the molten material moves outwards it pushes against the wall of the rim of the ablation crater, this solid barrier tends to retain the highly mobile melt. According to Markilie et al. the fiber ejection process corresponds to a breach of this retention process at some “weak point” on the rim allowing some hot liquid to be entirely ejected from the crater [30]. The ejected liquid moves rapidly through a very steep (downward) temperature gradient in its way out of the crater and therefore quickly starts to solidify. The NWs/fibers result from the solidification process of energetic molten droplets and their trails formed from the liquid streams ejected from the rim of the ablation crater. The trails which correspond to the NWs/fibers form due to the rapidly increasing viscous drag generated by the dropping temperature of the expelled liquid [29, 32]. Bulbous heads attached to long trails corresponds to the morphology that we routinely observe in many NWs (Additional file 1: Figure S3).

The NW/fiber synthesis process has been observed for a wide range of pulse widths (from 150 fs to 10 μs), powers (from 2 to 600 W) and pulse frequencies (single shots to 12.4 MHz). Our laser has a pulse width of 8 ns which falls within the tested range, on the other hand our power is much greater (0.31 GW) and the frequency is low (10 Hz) compared to most of the works reported. This has a beneficial effect in terms of ablation since more power is delivered to the target in each pulse. Indeed, we see large craters in our ablated pellets that show the evidence of a high degree of melting.

The formation of the decorating nanoparticles is also triggered by the ablation process. Fazio et al. observed the formation of Au and Ag nanoparticles formed by laser ablation in an Ar atmosphere (which matches our experimental situation). The NPs form in the ablation plume and are subsequently deposited in a self-organized manner over solid substrates placed directly in front of the target [35]. The same process of NP formation followed by deposition has been observed in ablated Au and Ag surfaces (among other metals) [36]. In the latter work the NPs gave rise to multiple re-colorations of the metallic surfaces. In the case of Ag, for instance, various NP sizes (from less than 10 nm up to over 30 nm) and interparticle deposition distances gave rise to yellow surfaces. Our ablated pellets show similar coloration effects. Before ablation, the B_2_O_3_-based pellets are white or with a very pale pink, pale yellow or slightly greenish coloration depending on whether they contain small amounts of Au, Ag or MoO_3_ respectively. After ablation the ablated areas of pellets with Au, Ag or MoO_3_ become bright red/pink, yellow or blue/purple. Furthermore, the color of some parts of the ablated pellets corresponds closely to the color of the decorated NWs produced from them.

Thus, the formation of decorated NWs can be explained in terms of the NP formation process proposed by Fazio et al. in conjunction with the mechanism for the formation of NWs/fibers explained above [35]. In short, the laser pulses locally melt the target and produce an ablation plume where the NPs are formed. Simultaneously, the highly mobile molten material is pushed towards the edge of the ablation crater by the plume’s pressure. There it gets ejected in the form of jets made of energetic droplets with long trails that form due to viscous drag. The jets rapidly solidify as they are expelled from the irradiated area forming NWs/fibers. The NWs/fibers collect NPs that get attached to their surface as they abandon the limits of the ablation plume where solidification is possible. Large numbers of NPs get deposited on the surface of the ablated target thus giving it its distinct coloration that matches that of the decorated NWs.

Although our work strongly focuses on developing a simple synthesis route for decorated NWs since the vast majority of the available methods are cumbersome and time consuming we can nevertheless say some words on possible applications for the NWs here produced. For instance, applications where decorated NWs are used as electrical components such as supercapacitors or structures with enhanced plasmonic resonances rely heavily on the mismatch between the electrical conductivities of the component parts, i.e. the metallic nanoparticles and the dielectric nanowires. If the nanowires are sufficiently insulating then charge trapping is favored in the metallic nanoparticles. Our NWs are chiefly made of B_2_O_3_ in various combinations with MgO, TiO_2_, MoO_3_ and Al_2_O_3_, these oxides have band gaps of 7 eV [37], 6.3 eV [38], 3.3–3.7 eV [39], 3.67 eV [40] and 5.1–8.8 eV [41] respectively. They starkly contrast with the zero bandgap structures of the noble metals forming the decorating nanoparticles of the NWs. Thus, we expect a significant bandgap mismatch could enhance the capacitance at the metal–dielectric interface formed between the nanoparticles and the NWs [23]. Additionally, the conductivity of the hybrid nanostructure in comparison to the bare NWs due to the formation of a tunable depletion layer at the metal–oxide interface [6]. Furthermore, it is known that Au nanoparticles show enhanced catalytic activity when supported on various metal oxides as it has been seen in the oxidation of CO and saturated hydrocarbons [42] and in the synthesis of boron nanowires covered by a thin boron suboxide layer [43]. Therefore, it is reasonable to expect that the as-produced NWs decorated with Au nanoparticles could show enhanced oxidative catalytic properties.

Finally, our results show that the addition of a pure metal in the pellet and therefore the presence of metallic NPs in the final products increases the yield of the NWs (Fig. 3). If the metal is absent the yield is low and the NWs are bare (as expected). This suggests that the NPs have some catalyzing effect in the production of the NWs that is different from the traditional VLS mechanism. However, at this stage we are uncertain so as to what exactly that mechanism might be. This is an aspect that needs further research and we hope to study it in the future.

## Conclusions

We have designed a room temperature single-step laser ablation technique to produce macroscopic amounts of a wide variety of decorated NWs as well as some bare nanowires with different superficial morphologies. The simplicity of the technique stands in stark contrast to the multi-step techniques that are usually implemented to synthesize decorated nanowires. The versatility of the technique has been proven by managing to produce 16 different types of NWs, however, the full extent of its versatility remains untapped since a more exhaustive combination of compounds can be tried. The versatility of the method is explained in terms of the physical processes taking place in the ablation crater. The NWs form due to the filamentary ejection of molten material from the crater which rapidly solidifies while simultaneously collecting NPs. The as-produced NWs have different morphologies and distinctive colorations, which signal to their equally distinctive optical properties. These type of decorated nanowires (semiconducting and/or dielectric NWs decorated with metallic NPs) have multiple tested applications, from sensing devices to charge storage and more. For these reasons we believe that this synthesis method could be attractive.

## Additional file

**Additional file 1.** Additional table and figures.

## Abbreviations

NPs: nanoparticles
NWs: nanowires
VLS: vapor–liquid–solid
SEM: scanning electron microscope
TEM: transmission electron microscope
VSS: vapor–solid–solid
