# Supplementary material for: Room temperature single-step synthesis of metal decorated boron-rich nanowires via laser ablation
Source: Nano Converg. 2019 May 8;6:14. doi: 10.1186/s40580-019-0185-2 (PMC6504969; doi:10.1186/s40580-019-0185-2)
Supplement: Supplementary file 1 — Additional file 1. Additional table and figures. [file 40580_2019_185_MOESM1_ESM.docx]

**Additional information.**

| **Chemical** | **Purity (%)** | **Provider** |
| --- | --- | --- |
| B_2_O_3_ | 99.98 | Alfa Aesar |
| MgO | 99.99 | Alfa Aesar |
| MoO_3_ | 99.5 | Alfa Aesar |
| TiO_2_ | 99.7 | Sigma Aldrich |
| ZnO | 99.999 | Puratronic |
| Al_2_O_3_ | 99.99 | Sigma Aldrich |
| Au | 99.96 | Alfa Aesar |
| Ag | 99.99 | Sigma Aldrich |
| Cu | 99.999 | Sigma Aldrich |
| Pd | 99.9 | Sigma Aldrich |
| Mo | 99 | Sigma Aldrich |
| Ge | 99.999 | Sigma Aldrich |
| Co | 99.8 | Alfa Aesar |

**Table S1. List of chemicals, purities and providers.**


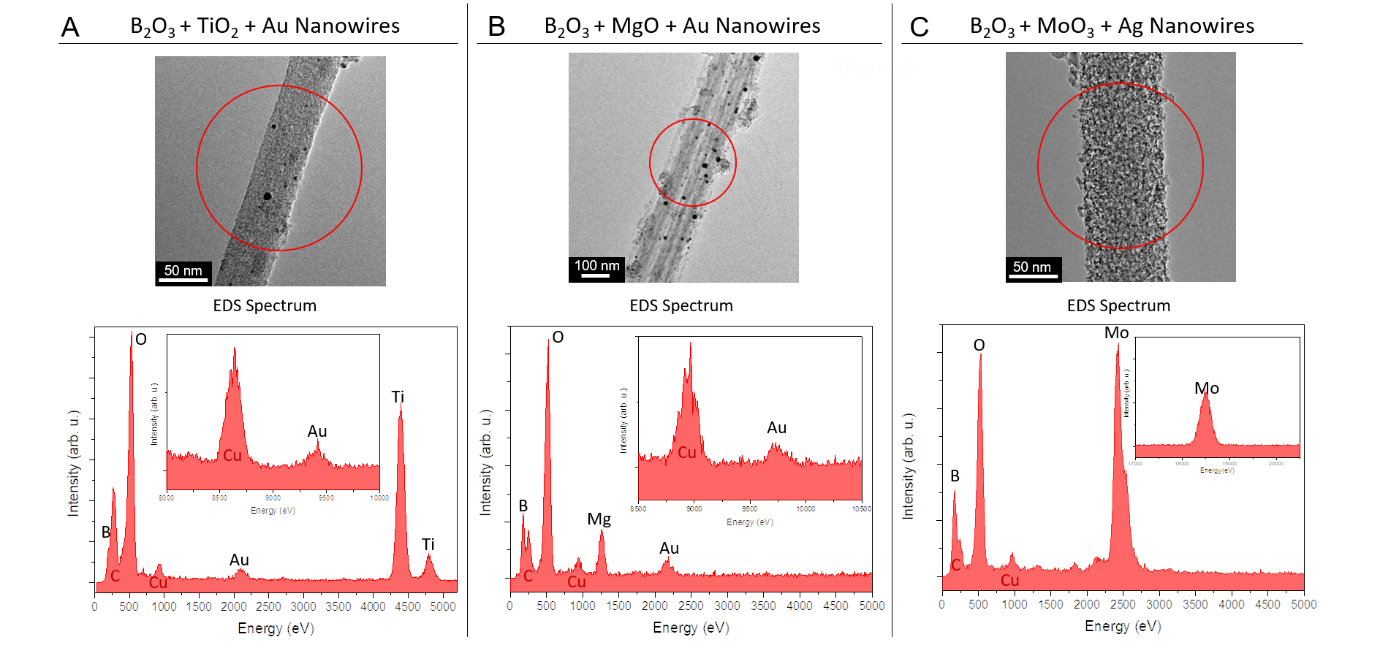


**Figure S1**. **EDS spectra of various types of nanowires. A, B** and **C** show TEM micrographs and EDS spectra of selected nanowires made of B_2_O_3_: TiO_2_: Au, B_2_O_3_:MgO:Au and B_2_O_3_: MoO_3_: Ag respectively. Images before and after EDS collection are presented in each case as well as the corresponding EDS spectrum right below the micrographs. The sampled regions are marked by a red circle in each case. Each EDS spectrum has an inset corresponding to the high energy region of the spectrum in question. The main peaks of interest are signaled with the chemical element written in black letters while other peaks coming from the grid or impurities are marked with dark red letters.


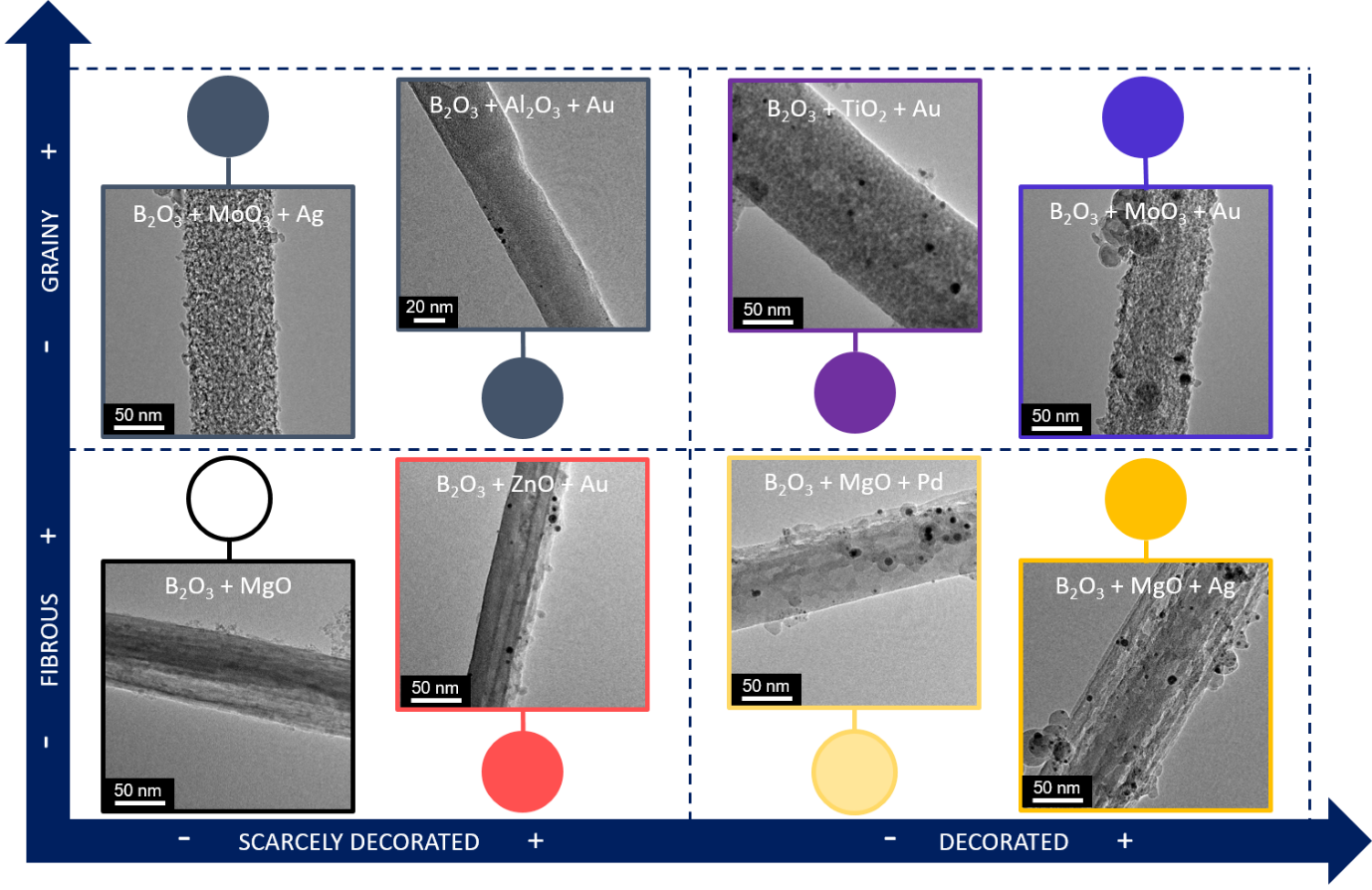


**Figure S2**. **Schematic arrangement of various types of nanowires according to their properties.** Representative TEM micrographs of eight different types of nanowires are arranged according to two main properties: how richly they are decorated with metallic nanoparticles and whether they have a fibrous texture or a coarser surface. The qualitative position of each type of nanowire respective to both scales is marked by a colored circle. The color of the circle closely matches the color of the nanowires when seen in macroscopic quantities. Nanowires placed more to the left of the X axis have less nanoparticles decorating them than those placed more towards the right side. Nanowires on the lower extreme of the Y axis are more fibre-like than those closer to the higher extreme which have a grainier texture. For example, nanowires made out of targets with B_2_O_3_:MgO belong to the higher left corner of the lower left quadrant because they tend to be rather smooth and it is possible to find Au particles on them although comparatively few with respect to other nanowires. On the other hand, B_2_O_3_:MoO_3_: Au are on the higher right corner of the higher right quadrant because they are very grainy and decorated with Au nanoparticles.


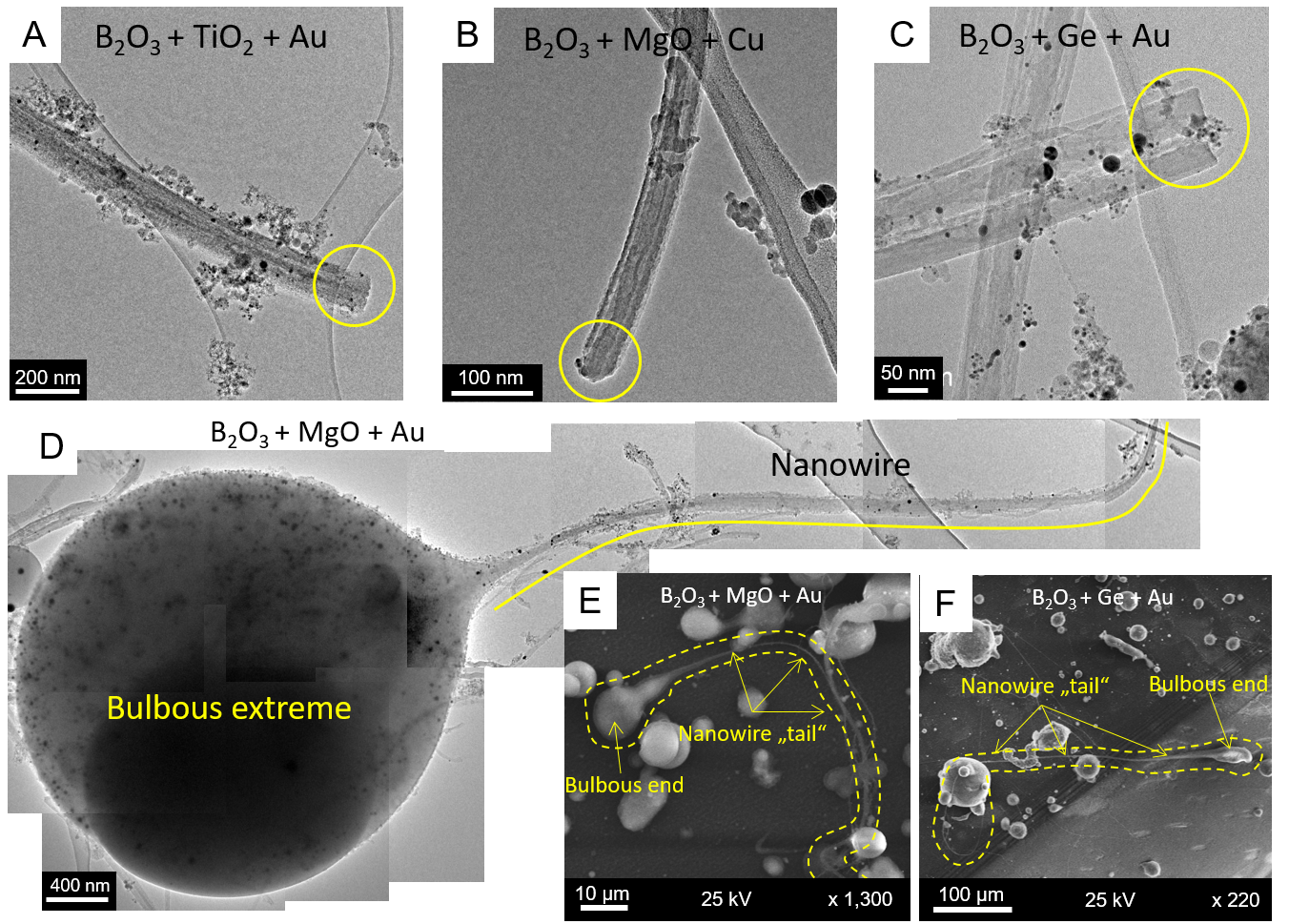


**FigureS3. Tips of the nanowires. A to C.** TEM micrographs of the tips of nanowires of various compositions (marked with yellow circles in each micrograph). None of the investigated nanowires had a metallic nanoparticle at its tip, i.e. the typical piece of evidence hinting at the vapour-liquid-solid mechanism as the phenomenon driving the growth process. An alternative possibility is that all the observed nanowires happened to be broken and we just observed the broken extreme. **D**. A nanowire with a large bulbous end at one of its extremes. This type of ending was common irrespective of the chemical composition of the nanowires. Another example of this morphology can be seen in the SEM image in **E**. **F.** Another example of a nanowire with a bulbous end at one of its extremes, this nanowire was obtained by ablating targets with B_2_O_3_: Ge: Au.
